# Molecular and phenotypic characterization of efflux pump and biofilm in multi-drug resistant non-typhoidal *Salmonella* Serovars isolated from food animals and handlers in Lagos Nigeria

**DOI:** 10.1186/s42522-021-00035-w

**Published:** 2021-02-09

**Authors:** Elizabeth Tolulope Olubisose, Abraham Ajayi, Adeyemi Isaac Adeleye, Stella Ifeanyi Smith

**Affiliations:** 1 Department of Microbiology, University of Lagos Akoka, Lagos, Nigeria; 2 Molecular Biology and Biotechnology Department, Nigerian Institute of Medical Research Lagos, Lagos, Nigeria; 3 Mountain Top University, Lagos-Ibadan Expressway, Ibafo, Ogun State Nigeria

**Keywords:** Efflux pump, Biofilm, Antibiotics, Multidrug resistance, *Salmonella*

## Abstract

**Background:**

Multidrug resistance efflux pumps and biofilm formation are mechanisms by which bacteria can evade the actions of many antimicrobials. Antibiotic resistant non-typhoidal *Salmonella* serovars have become wide spread causing infections that result in high morbidity and mortality globally. The aim of this study was to evaluate the efflux pump activity and biofilm forming capability of multidrug resistant non-typhoidal *Salmonella* (NTS) serovars isolated from food handlers and animals (cattle, chicken and sheep) in Lagos.

**Methods:**

Forty eight NTS serovars were subjected to antibiotic susceptibility testing by the disc diffusion method and phenotypic characterization of biofilm formation was done by tissue culture plate method. Phenotypic evaluation of efflux pump activity was done by the ethidium bromide cartwheel method and genes encoding biofilm formation and efflux pump activity were determined by PCR.

**Results:**

All 48 *Salmonella* isolates displayed resistance to one or more classes of test antibiotics with 100% resistance to amoxicillin-clavulanic acid. Phenotypically, 28 (58.3%) of the isolates exhibited efflux pump activity. However, genotypically, 7 (14.6%) of the isolates harboured *acrA*, *acrB* and *tolC*, 8 (16.7%) harboured *acrA*, *acrD* and *tolC* while 33 (68.8%) possessed *acrA*, *acrB*, *acrD* and *tolC*. All (100%) the isolates phenotypically had the ability to form biofilm with 23 (47.9%), 24 (50.0%), 1 (2.1%) categorized as strong (SBF), moderate (MBF) and weak (WBF) biofilm formers respectively but *csgA* gene was detected in only 23 (47.9%) of them. Antibiotic resistance frequency was significant (*p* < 0.05) in SBF and MBF and efflux pump activity was detected in 6, 21, and 1 SBF, MBF and WBF respectively.

**Conclusion:**

These data suggest that *Salmonella* serovars isolated from different food animals and humans possess active efflux pumps and biofilm forming potential which has an interplay in antibiotic resistance. There is need for prudent use of antibiotics in veterinary medicine and scrupulous hygiene practice to prevent the transmission of multidrug resistant *Salmonella* species within the food chain.

## Background

*Salmonella* are motile rod-shaped (bacilli) Gram negative bacteria of the family Enterobacteriaceae. Non-typhoidal *salmonella* serovars mostly cause gastroenteritis, bacteremia, and focal infection. Ingestion of contaminated animal products, such as poultry, pork, and other meats is a major route of transmission in humans. Direct contact is also a potential route of transmission in animals such as chicks, ducklings and other animals that may also transmit the bacterium to humans [1].

Antimicrobial resistance remains a global public health concern threatening the effectiveness of antibacterial therapy. Different variants of bacterial pathogens isolated globally have now become multidrug resistant. Antibacterial resistance occurs by numerous mechanisms including enzymatic inactivation, modification of drugs, drug target alteration or protection, and efflux of drugs through efflux pumps. Antibiotic resistance has been seen in both typhoidal and non-typhoidal serovars [2]. Food animals and handlers contribute largely to the spread of zoonotic multidrug resistant non-typhoidal *Salmonella.* Efflux generally involves transportation of a substance out of the cell. Efflux pumps play an essential role in the physiology of bacteria by mediating the entry and extrusion of essential nutrients, metabolic waste and xenobiotics. Bacterial efflux systems generally fall into five classes, the major facilitator (MF) superfamily, the ATP-binding cassette (ABC) family, the resistance-nodulation-division (RND) family, the small multidrug resistance (SMR) family and the multidrug and toxic compound extrusion (MATE) family [3]. *Salmonella* has at least one MDR pump from each family with the exception of the SMR family of efflux pumps, all of the identified MDR efflux systems also exist in *E. coli* with the exception of MdsABC (mds-multidrug transporter for *Salmonella*) which is unique to *Salmonella* [4]. The best characterised of the RND pumps in *Salmonella* is AcrB and its tripartite complex AcrAB-TolC which has many different substrates making this efflux pump (and other RND homologues) a key mediator of multidrug resistance in Gram negative bacteria including many Enterobacteriaceae [5]. Another recently described family of transport protein is the Proteobacterial antimicrobial compound efflux (PACE) systems that is said to be considered across many Gram-negative pathogens including, *Klebsiella, Burkholderia, Salmonella, Pseudomonas* and *Enterobacter* species. They have been shown to mediate resistance to several antimicrobials including chlorhexidine, dequalinium, acriflavine, benzalkonium and proflavine [6]. Biofilm formation contributes largely to the resistance of bacteria to different classes of antimicrobials. They can act synergistically with efflux pumps leading to elevated levels of clinical significance. *Salmonella* biofilms are encased in a matrix largely composed of two major components; curli and cellulose. They are involved in many functions including adhesion, cell aggregation, environmental persistence and biofilm formation [7]. This study was aimed at evaluating the antibiotic resistance profile of NTS serovars as well as their biofilm forming and efflux pump activity potentials.

## Methods

### Study design and bacterial strains

This descriptive study was conducted in Lagos southwestern Nigeria. Forty eight NTS serovars isolated from food animals comprising 28 isolates from chicken, 3 from cattle, 9 from sheep and 8 from apparently healthy food handlers as previously reported [8].

### Antibiotic susceptibility testing

Antibiotic susceptibility testing (AST) was done according to the guidelines of the European committee on antimicrobial susceptibility testing [9] using the disc diffusion method. A loop full of a 24 h brain heart infusion broth culture of isolates were streaked on nutrient agar plate incubated for 24 h at 37 °C. One or two colonies were picked and emulsified in 5 mL of normal saline and adjusted to 0.5 McFarland standard. Using a sterile swab stick, bacterial suspensions were applied to the surface of Muller-Hinton agar (Oxoid, Basingstoke, UK) after which test antibiotic discs were applied and incubated at 37 °C for 24 h. Zones of inhibition were measured and interpreted as Resistance (R), Intermidiate (I) and Sensitive (S) with break points of 22–19 for ceftazidime (30 μg), 19–19 for cefuroxime (30 μg), 17–14 for gentamicin (10 μg), 17–17 for cefixime (5 μg), 24–22 for ofloxacin (5 μg), 19–19 for amoxilin _+_ clavulanic acid (30 μg), 11–11 for nitrofurantion (300 μg), 25–22 for ciprofloxacin (5 μg), 15–11 for tetracycline (30 μg), 19–15 for nalidixic acid (30 μg) and 22–17 for Imipenem (30 μg) (range implies the values between sensitivity ≤ and resistance >). AST was done in duplicates and *Escherichia coli* ATCC 25922 was used as quality control organism.

### Phenotypic characterization of biofilm formation

#### Tissue culture plate method

Biofilm formation was evaluated by the tissue plate method according to Christensen et al. [10] and Cavant et al. [11] with slight modifications. Isolates were inoculated into brain heart infusion broth (Oxoid, Basingstoke, UK) supplemented with 2% of sucrose and incubated for 18 h at 37 °C. A one in 100 dilution of the culture was made with fresh sterile brain heart infusion broth and 0.2 mL was dispensed into individual wells of a 96 well tissue culture plate. Sterile broth served as negative control and *Salmonella Typhimurium* 14028 was inoculated into separate wells as positive control. Incubation was done at 37 °C for 24 h. After incubation content of each well was gently removed by tapping the plates. The wells were washed four times with 0.2 mL of phosphate buffer saline (PBS pH 7.2) to remove free-floating planktonic bacteria. The plates were then stained with crystal violet (0.1% w/v) and allowed to stay for 45 min. Excess stain was rinsed off by washing with deionized water thrice and plates were allowed to air dry. Crystal violet incorporated by the adherent cells was solubilized by adding 200 μL of 33% glacial acetic acid (Merck, Darmstadt, Germany). The optical density (OD) of each well was determined with an Emax® Plus Microplate Reader (Molecular Devices San Jose, CA) at wave length 570 nm. The experiment was performed in triplicates and repeated three times. Absorbance was calculated by subtracting the OD_570_ of control from that of the assays OD_570_ with mean value determined for each isolate. Data obtained was used to classify OD_570_ values < 0.120 as weak biofilm formers, values between 0.120–0.240 as moderate biofilm formers, and > 0.240 as strong biofilm formers.

#### Determination of efflux pump activity by Ethidium bromide cartwheel method

The ethidium bromide cartwheel method according to Martins et al. [12] was used in evaluating efflux pump activity in isolates. Approximately 10^6^ cells per mL of *Salmonella* isolates were streaked on Muller-Hinton agar plates containing 0 mg/L, 0.5 mg/L, 1 mg/l, 1.5 mg/L and 2 mg/L concentrations of EtBr and incubated at 37 °C for 24 h. After incubation, the plates were examined under UV light. Fluorescence of isolates at different concentrations of EtBr were noted. Isolates without fluorescence indicated active efflux pump activity while those that fluoresced lacked efflux pump activity.

#### Detection of efflux pump and biofilm encoding genes

Genomic DNA of isolates was extracted according to the method of Kpoda et al. [13] Four efflux pump encoding genes (*acrA, acrB, acrD tolC*) and one biofilm formation encoding gene (*csg A*) were assayed for by monoplex PCR targeting specific primers listed in Table 1. A 20 μL PCR reaction was used which contained 10.8 μL nuclease free water, 0.6 μL forward primer, 0.6 μL reverse primer, 4 μL DNA template and 4 μl of 5X PCR Master Mix (7.5 mM MgCl_2_, 1 Mm dNTPs, 0.4 M Tris-HCl, 0.1 M (NH_4_)_2_SO_4_, 0.1% Tween-20, FIREPol DNA Polymerase) (Solis BioDyne Estonia). PCR was carried out in an Eppendorf thermal cycler (Eppendorf AG, Hamburg, Germany) with PCR programming conditions of initial DNA denaturation at 95 °C for 5 min, followed by 30 cycles of denaturation at 95 °C for 30 s, annealing at 57 °C for *csgA*, 56 °C for *acrA*, 54 °C for *tolC* and 55 °C for *acrB* and 51 °C for *acrD* for 30 s and extension at 72 °C for 2 min, followed by a final extension at 72 °C for 10 min. PCR products were electrophoresed at 100 V for 1 h in 2% agarose gel stained with ethidium bromide and visualized under ultraviolet trans-illuminator (Cleaver Scientific Ltd). A 100 bp DNA ladder (Solis Biodyne, Estonia) was used as a molecular weight marker.

**Table 1:** List of Primers Used in Targeting Genes Encoding Efflux Pump and Biofilm Forming Ability

Primer	Sequence (5′-3′)	Product Size (bp)	Reference
*acrA*-FW *acrA* -RV	CTCTCAGGCAGCTTAGCCCTAA AACAGTCAAAACTGAACCTCTGCA	106	[14]
*acrB*-FW *acrB*-RV	GGTCGATTCCGTTCTCCGTTA ATGACGTTTACTTCCAGGTAG	104	[14]
*acrD*-FW *acrD*-RV	AATTGTGCGTGAAGCGGT GCTACAGCGCCATAGTAA	100	[15]
*tolC*-FW *tolC*-RV	AAGCCGAAAAACGCAACCT GATGGTCACTTACCGACTCTG	100	[14]
*csgA*-FW *csgA*-RV	GCAATCGTATTCTCCGGTAG GATGAGCGGTCGCGTTGTTA	418	[16]

### Data analysis

Graphics and data analysis were performed by Microsoft Excel (Microsoft Cooperation, 2013 USA) and GraphPad Prism version 8.0.2 (GraphPad Software Inc. USA). One way ANOVA test was used to determined association between variables. Statistical significance was considered for *p* < 0.05.

## Results

All 48 (100%) *Salmonella* isolates were susceptible to nitrofurantion and imipenem. They all (100%) were however, resistant to amoxicillin-clavulanic acid. Forty five (93.8%) of the isolates were resistant to ceftazidime and cefuroxime, 13 (27.1%) were resistant to cefixime, 23 (47.9%) were resistant to gentamicin, 15 (31.3%) were resistant to ofloxacin, 19 (39.6%) were resistant to ciprofloxacin, 33 (68.8%) were resistant to tetracycline and 20 (41.7%) were resistant to nalidixic acid as shown in Table 2. Twenty five (52.1%) of the isolates were multidrug resistant (MDR).

**Table 2:** Percentage Susceptibility of Isolates to Various Classes of Antibiotics

Class of Antibiotics	Antibiotics	Sensitive (%)	Resistant (%)
Beta-lactam	Amoxicillin-clavulanic acid	0	100
	Ceftazidime	6.2	93.8
	Cefuroxime	6.2	93.8
	Cefixime	72.9	27.1
Carbapenem	Imipenem	100	0
Aminoglycoside	Gentamicin	52.1	47.9
Quinolone	Ciprofloxacin	60.4	39.6
	Ofloxacin	68.7	31.3
	Nalidixic acid	58.3	41.7
Tetracycline	Tetracycline	31.2	68.8
Others	Nitrofurantoin	100	0

**Fig. 1:**
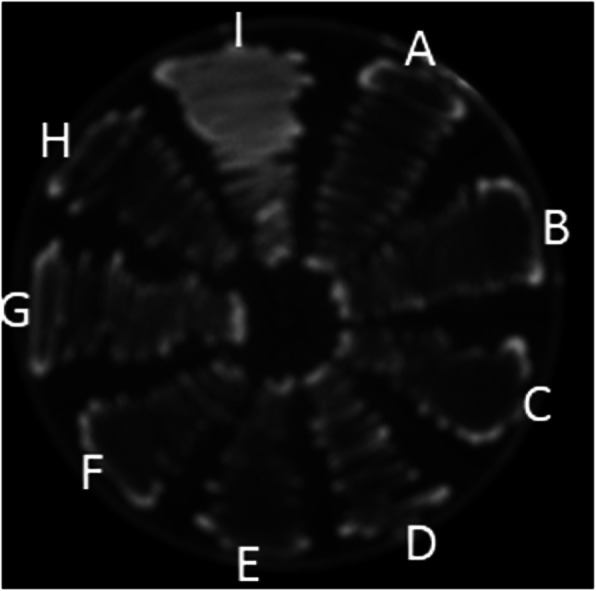
Efflux pump activity of *Salmonella* isolates determined by the ethidium bromide cartwheel method. Isolates A, B, C, D, E, F, G, H are positive for efflux pump activity as they did not fluoresce under UV light. Isolate I lacks efflux pump activity and fluoresced because of ethidium bromide retention

**Fig. 2:**
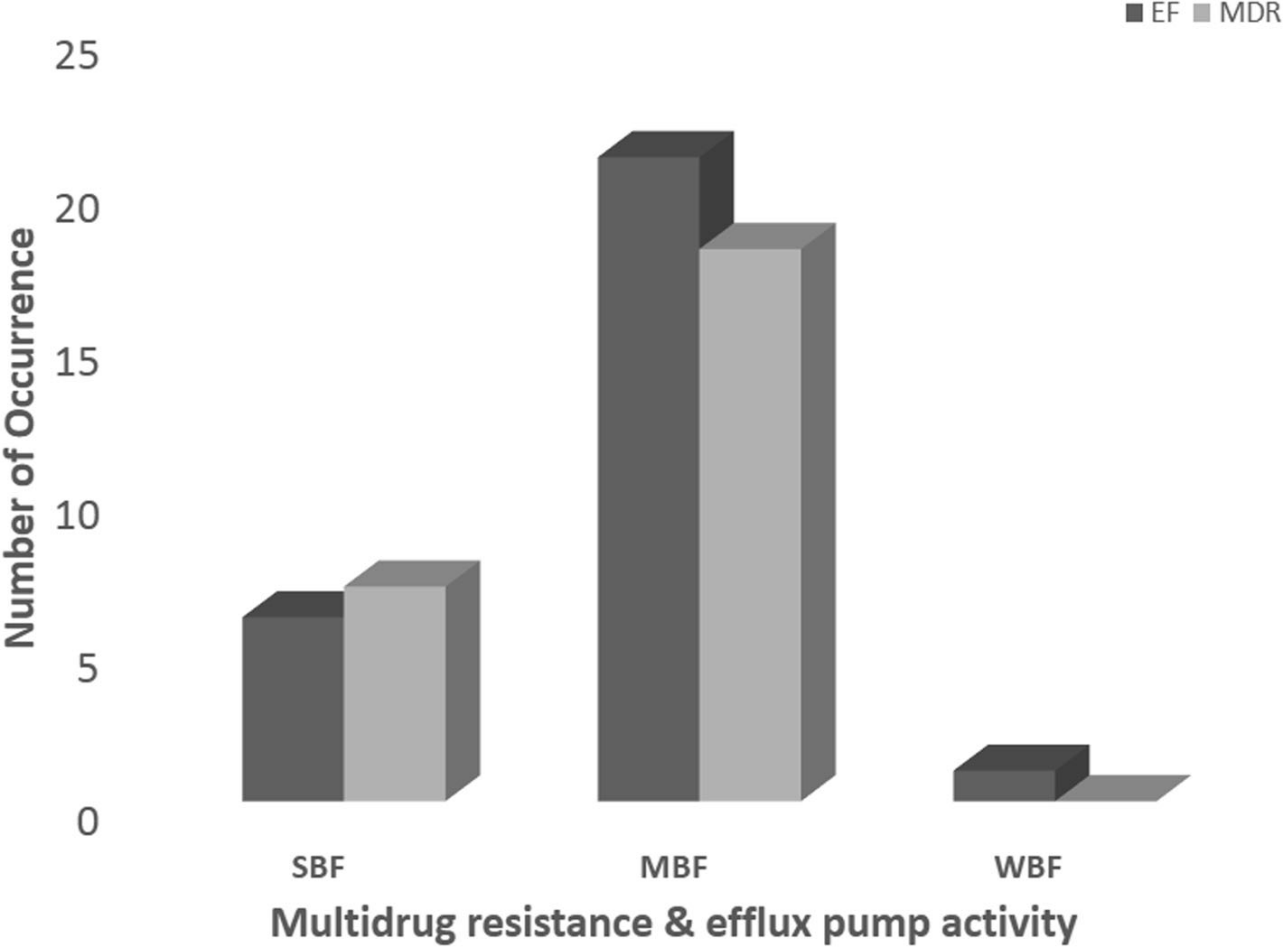
Occurrence of multidrug resistance and efflux pump activity of biofilm forming isolates. SBF: Strong biofilm formers, MBF: Moderate biofilm former, WBF: Weak biofilm former, EF: Efflux pump activity

**Fig. 3:**
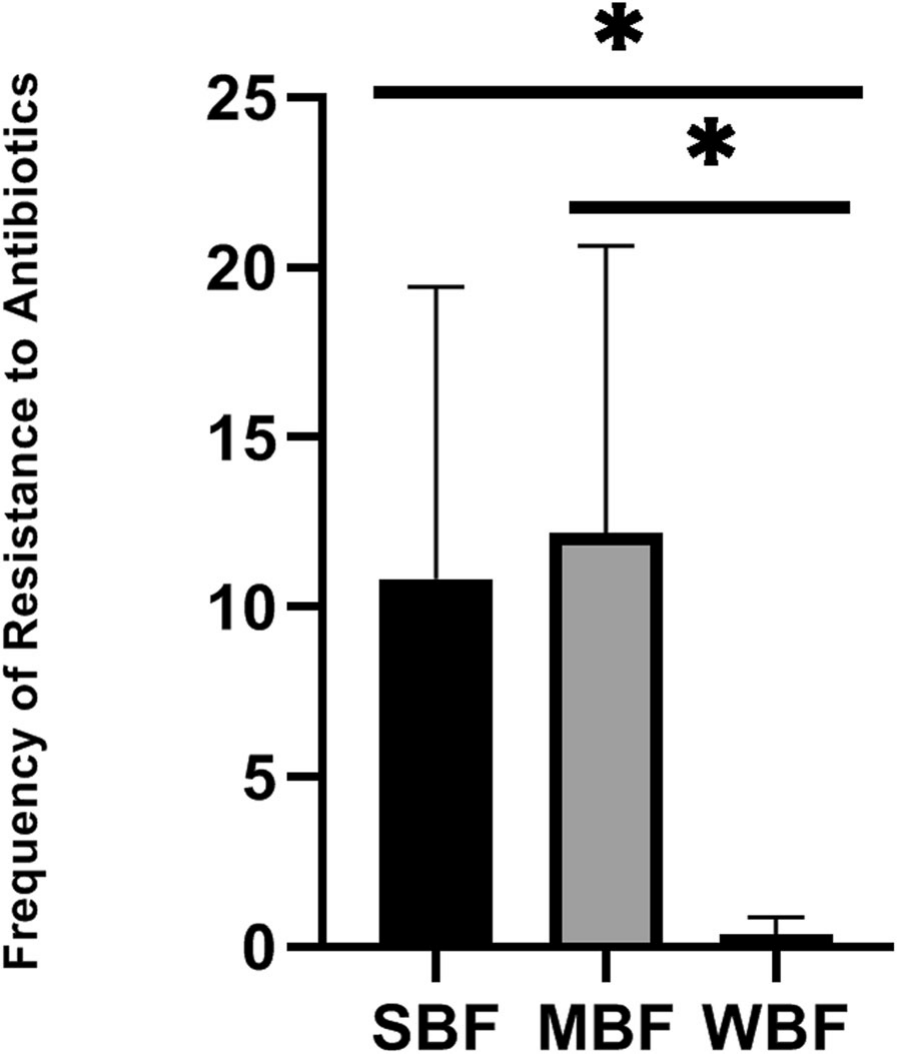
Biofilm forming potential and antibiotic resistance frequency. Values are expressed as means±SD, *P < 0.05

**Fig. 4:**
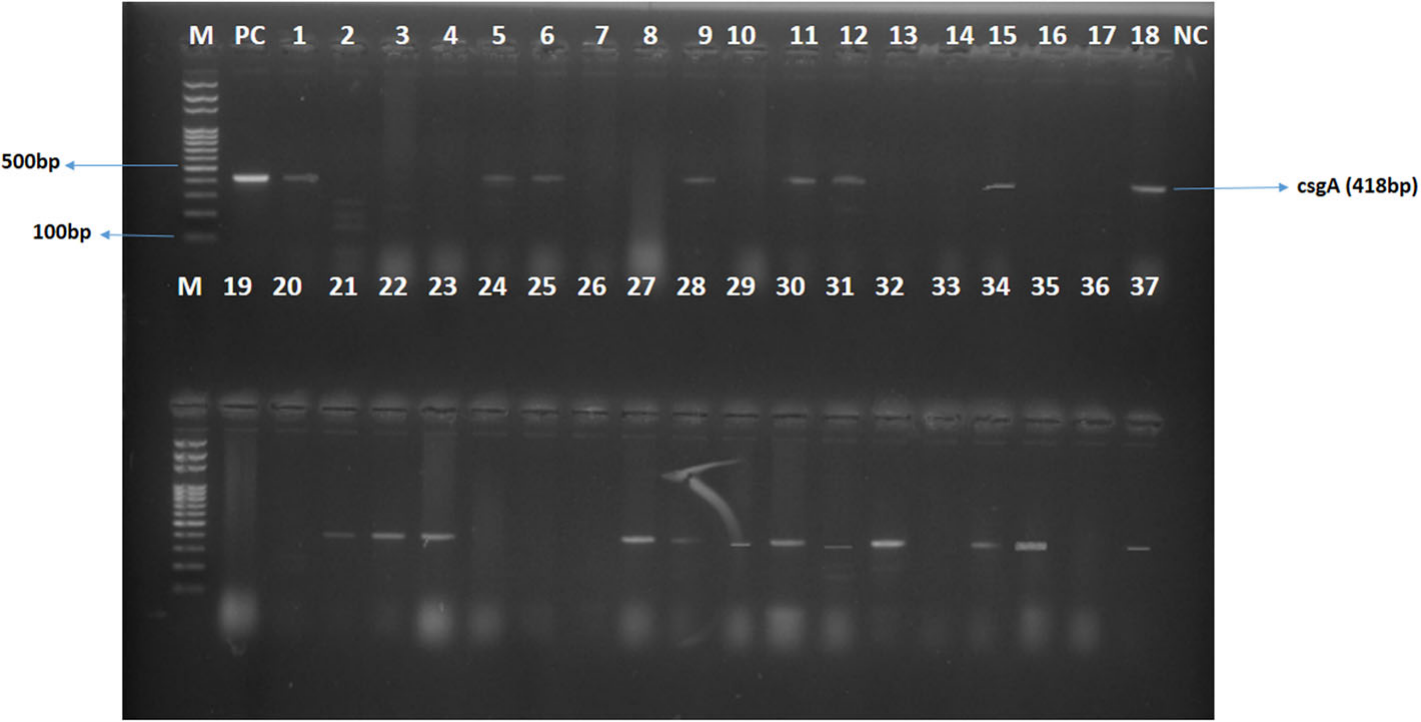
Agarose gel image of PCR products showing bands for *csgA* (418 bp). Lane M: Molecular maker, Lane 1, 5, 6, 9, 11, 12, 15, 18, 21, 22, 23, 27, 28, 29, 30, 31, 32, 34, 35 and 37 are positive for *csgA*

All *Salmonella* isolates were biofilm formers and 23 (47.9%), 24 (50.0%) and 1 (2.1%) of the isolates were strong (SBF), moderate (MBF) and weak (WBF) biofilm formers respectively. Also 28 (58.3%) of the isolates phenotypically displayed efflux pump activity as they did not fluoresce under UV light since they did not retain ethidium bromide within their cells as shown in Fig. 1. Of the 25 MDR isolates 7 were SBF and 18 were MBF while the WBF was not MDR. Furthermore, efflux pump activity was detected in 6, 21, and 1 SBF, MBF and WBF respectively as shown in Fig. 2. It was also observed that antibiotic resistance frequency was significant (*p* < 0.05) in SBF and MBF as shown in Fig. 3. Although all the isolates had the ability to form biofilm, *csgA* gene was only detected in 23 (47.9%) of the isolates as shown in Fig. 4.

*acrA*, *acrB* and *tolC* were detected in 7 (14.6%) of the isolates, *acrA*, *acrD* and *tolC* were detected in 8 (16.7%) of the isolates while 33 (68.8%) possessed all four genes *acrA*, *acrB*, *acrD* and *tolC*. Although majority of isolates from chicken, sheep and human harboured all four efflux pump genes, some did not phenotypically exhibit efflux pump activity as shown in Table 3.

**Table 3:** Source of *Salmonella* Serovars, their Biofilm Forming Potential, Efflux Pump Activity and Efflux Pump Genotype

S/N	Code	^**a**^Source	^**b**^Serovar	Biofilm Potential	Efflux Pump Activity	Efflux Pump Genotype
1	A1	Chicken	*S.* Ebrie	Moderate	Positive	*acrA, acrB, tolC*
2	A2	Chicken	*S.* Budapest	Moderate	Negative	*acrA, acrD, tolC*
3	A3	Chicken	*S*. Muenster	Moderate	Positive	*acrA, acrB, acrD, tolC*
4	A4	Chicken	*S.* Dabou	Strong	Positive	*acrA, acrB, acrD, tolC*
5	A5	Chicken	*S.* Budapest	Strong	Positive	*acrA, acrB, acrD, tolC*
6	A6	Chicken	*S*. Tennyson	Moderate	Positive	*acrA, acrB, acrD, tolC*
7	A7	Chicken	*S.* Budapest	Moderate	Positive	*acrA, acrB, acrD, tolC*
8	A8	Chicken	*S.* Brandenburg	Moderate	Positive	*acrA, acrB, acrD, tolC*
9	A9	Chicken	*S.* Anecho	Strong	Positive	*acrA, acrB, tolC*
10	A10	Chicken	*S*. Minna	Moderate	Positive	*acrA, acrB, acrD, tolC*
11	A11	Chicken	*S.* Budapest	Moderate	Negative	*acrA, acrD, tolc*
12	A12	Chicken	*S.* Budapest	Moderate	Positive	*acrA, acrB, acrD, tolC*
13	A13	Chicken	*S.* Budapest	Strong	Negative	*acrA, acrD, tolC*
14	A14	Chicken	*S.* Budapest	Moderate	Positive	*acrA, acrB, tolC*
15	A15	Chicken	*S.* Budapest	Strong	Negative	*acrA, acrD, tolC*
16	A16	Chicken	*S.* Budapest	Moderate	Positive	*acrA, acrD, tolC*
17	A17	Chicken	*S.* Agodi	Strong	Negative	*acrA, acrB, acrD, tolC*
18	A18	Chicken	*S.* Budapest	Moderate	Positive	*acrA, acrB, acrD, tolC*
19	A19	Chicken	*S*. Essen	Strong	Negative	*acrA, acrB, acrD, tolC*
20	A20	Chicken	*S.* Budapest	Moderate	Positive	*acrA, acrB, acrD, tolC*
21	A21	Chicken	*S.* Anecho	Strong	Positive	*acrA, acrB, acrD, tolC*
22	A22	Chicken	*S.* Agodi	Strong	Negative	*acrA, acrD, tolC*
23	A23	Chicken	*S*. Kaapstad	Moderate	Positive	*acrA, acrB, acrD, tolC*
24	A24	Chicken	*S.* Anecho	Strong	Negative	*acrA, acrB, acrD, tolC*
25	A25	Chicken	*S.* Ealing	Strong	Negative	*acrA, acrB, acrD, tolC*
26	A26	Chicken	*S.* Wichita	Moderate	Negative	*acrA, acrB, acrD, tolC*
27	A27	Chicken	*S.* Budapest	Moderate	Positive	*acrA, acrB, acrD, tolC*
28	A28	Chicken	*S.* Budapest	Strong	Negative	*acrA, acrB, acrD, tolC*
29	S1	Sheep	*S.* Chomedey	Strong	Positive	*acrA, acrB, acrD, tolC*
30	S2	Sheep	*S*. Dahra	Moderate	Positive	*acrA, acrB, acrD, tolC*
31	S3	Sheep	*S*. Yovokome	Moderate	Positive	*acrA, acrB, acrD, tolC*
32	S4	Sheep	*S*. Sculcoates	Strong	Positive	*acrA, acrB, tolC*
33	S5	Sheep	*S.* Berlin	Strong	Negative	*acrA, acrB, acrD, tolC*
34	S6	Sheep	*S*. Essen	Strong	Negative	*acrA, acrB, acrD, tolC*
35	S7	Sheep	*S*. Livingstone	Strong	Negative	*acrA, acrB, acrD, tolC*
36	S8	Sheep	*S.* Mura	Moderate	Positive	*acrA, acrB, acrD, tolC*
37	S9	Sheep	*S.* Orion	Strong	Negative	*acrA, acrB, acrD, tolC*
38	H1	Human	*S*. Tyhpimurium	Moderate	Positive	*acrA, acrB, acrD, tolC*
39	H2	Human	*S*. Portland	Moderate	Positive	*acrA, acrB, acrD, tolC*
40	H3	Human	*S*. Limete	Strong	Negative	*acrA, acrB, tolC*
41	H4	Human	*S*. Takoradi	Weak	Positive	*acrA, acrD, tolC*
42	H5	Human	*S*. Chagoua	Strong	Negative	*acrA, acrB, acrD, tolC*
43	H6	Human	*S*. Huettwillen	Strong	Negative	*acrA, acrB, acrD, tolC*
44	H7	Human	*S*. Paratyphi C	Strong	Negative	*acrA, acrB, acrD, tolC*
45	H8	Human	*S*. Mowanjum	Moderate	Positive	*acrA, acrB, acrD, tolC*
46	CO1	Cow	*S*. Budapest	Moderate	Positive	*acrA, acrD, tolC*
47	CO2	Cow	(43,:g, z_62_:enx) II	Moderate	Positive	*acrA, acrB, tolC*
48	CO3	Cow	*S*. Somone	Strong	Negative	*acrA, acrB, tolC*

^**a, b**^Source of *Salmonella* isolates and serotypes previously reported in Ajayi et al. [8]

## Discussion

In this study all *Salmonella* isolates were sensitive to imipenem and nitrofurantoin indicating a low pressure on the use of these antibiotics. This is in line with the study of Akinyemi et al. [17] who reported a 100% sensitivity of *Salmonella* spp. isolated from different sources in Nigeria to imipenem. Similarly, Albert et al. [18] in their findings reported a total sensitivity of NTS isolated from blood stream infection in Kuwait to imipenem. However, antibiotic resistance to various classes of antibiotics was recorded in this study with 52.1% of MDR isolates. *Salmonella* serovars showed high resistance to third generation cephalosporins where 93.8% were resistant to ceftazidime and cefuroxime and 27.1% were resistant to cefixime. This is in line with the report of Musa et al. [19] who reported multiple resistance patterns of *Salmonella* species isolated from human stool samples and raw meat to cefuroxime, ceftazidime and ceftriaxone in Niger state Nigeria. In a previous study, Akinyemi et al. [20] in Lagos Nigeria also reported increase in *Salmonella* spp. resistance to third generation cephalosporin. Resistance of NTS serovars to quinolones in this study was also common. Twenty (41.7%), 15 (31.3%) and 19 (39.6%) *Salmonella* serovars were resistant to nalidixic acid, ofloxacin and ciprofloxacin respectively. Quinolones most especially ciprofloxacin remains a drug of choice for the treatment of *Salmonella*. However the wide spread resistance to this antibiotic is worrisome. Katiyo et al. [21] in a study between 2004 and 2015 of NTS bacteremia in England revealed a high resistance to ciprofloxacin and nalidixic acid. All *Salmonella* isolates in this study had biofilm forming capability which comprised 23 (47.9%) of SBF, 24 (50%) of MBF and 1 (2.1%) of WBF. Of these biofilm formers 7 of the SBF and 18 of the MBF were MDR indicating the probable role of biofilm in mediating multidrug resistance as the only WBF was not MDR. In a similar study, Farahani et al. [22] reported a 34.5% prevalence of strong biofilm forming MDR *S. Enteritidis* isolated from poultry and clinical isolates. Furthermore, *csgA* gene was detected in 23 (47.9%) of *Salmonella* isolates in this study. *csgA* gene is known to facilitate biofilm formation in *Salmonella* species as it is part of the *csgBAC* operon that encodes the structural genes of curli fimbriae [23]. Efflux pumps are important mechanisms that mediate antibiotic resistance in *Salmonella*. The resistance-nodulation-division (RND) family of efflux pump to which the *acrAB-tolC* and *acrD* belong has been widely reported in *E. coli* and *Salmonella* spp. and is known to confer MDR [24]. In this study a combination of all four gene *acrA*, *acrB*, *acrD* and *tolC* were detected in 33 (68.8%) of the *Salmonella* isolates, while *acrA*, *acrB* and *tolC* were present in 7 (14.6%) and 8 (16.7%) harboured *acrA*, *acrD* and *tolC*. Of these isolates that possessed these genes, 28 (58.3%) comprising 6 SBF, 21 MBF and 1 WBF phenotypically exhibited efflux pump activity. The presence of these genes could be linked to the observed resistance profile of the isolates. In the report of Yamasaki et al. [25] it was detected that the overexpression of *acrD* resulted in increased drug resistance in *S.* Typhimurium. Similarly, Shen et al. [26] reported the involvement of AcrAB-TolC efflux pump system in mediating fluoroquinolone resistance in *Salmonella* serovars isolated from meat and human in China. The role of these efflux pumps transcends mediating antibiotic resistance, their role in biofilm formation and other physiological functions have been reported. Buckner et al. [27] reported the role of *acrD* efflux pump in the biology of *Salmonella* including virulence, basic metabolism and stress responses. Baugh et al. [28] also demonstrated a link between biofilm formation and efflux pump systems of *Salmonella*. Hence, the presence of these efflux pumps in *Salmonella* isolates as observed in this study bring to bear intrinsic mechanism explored by this pathogen in extruding extraneous materials including antibiotics and in maintaining viability.

## Conclusion

In this study several *Salmonella* serovars isolated from food animals and food handlers were multidrug resistant which would have been mediated by efflux pump activity and biofilm formation potentials which they possessed. Beyond mediating antibiotic resistance, biofilm formation by *Salmonella* spp. also enable them to be persistent in food processing environments hence promoting their transmission and colonization of multiple hosts. Therefore, food, personal and environmental hygiene is imperative with constant epidemiological surveillance to track and control the transmission of the pathogen within the food chain.

## Data Availability

Data sets used and analysed for this study are available from the corresponding author on reasonable request. All data generated or analysed during this study are also included in this published article.

## Abbreviations

EtBr: Ethidium bromide
MDR: Multidrug resistant
MBF: Moderate biofilm former
NTS: Non-typhoidal *Salmonella*
RND: Resistance nodulation division
SBF: Strong biofilm former
OD: Optical density
WBF: Weak biofilm former
